# A minimally invasive bipolar surgical approach for the treatment of patellar fracture using the tension-band wiring technique

**DOI:** 10.3389/fsurg.2022.955651

**Published:** 2022-11-11

**Authors:** Zhangxiong Lin, Yaoqing Chen, Hai Wang, Wenbin Lan, Yun Xie, Gui Wu

**Affiliations:** Department of Orthopedics, First Affiliated Hospital, Fujian Medical University, Fuzhou, Fujian

**Keywords:** minimally invasive surgical approach, patellar fracture, tension-band wiring, patella, knee scar

## Abstract

**Objective:**

Minimally invasive surgical techniques are becoming increasingly popular for the treatment of traumatic injuries. Although some minimally invasive techniques in the management of patellar fractures have been reported, the limited exposure in such methods may cause technical difficulties during surgery and restrict their wide application. In this context, this study aims to introduce a bipolar incision and assess the clinical outcomes of patellar fractures treated via this type of incision.

**Materials and methods:**

Patients who suffered patellar fractures and who received surgical treatment via bipolar incision between 2018 and 2020 in our hospital were retrospectively reviewed and included in this study. The clinical and radiological records of all patients were reviewed. A classification of the fractures was done and intraoperative parameters, Visual Analog Scale (VAS) score, knee range of motion, and the Hospital for Special Surgery (HSS) knee score of the patients were evaluated and summarized.

**Results:**

The study included 19 patients who met the inclusion criteria. All patellar fractures were operated through the minimally invasive bipolar surgical approach. The mean time of operation was 69.0 ± 8.5 min. The mean time to union was 12.8 ± 2.1 weeks. The average total knee range of motion was 131.8 ± 4.4°, and the average HSS score was 97.1 ± 2.6 at 1-year post-operation. No surgical-related complications were observed.

**Conclusions:**

The knee functional outcomes were favorable when patellar fractures were treated through the minimally invasive bipolar incision method. This bipolar surgical approach was found to be a feasible method for treating patellar fractures.

## Introduction

The patella is located anteriorly to the knee joint and plays a key role in increasing the moment arm of the extensor mechanism. Patellar fractures account for approximately 1% of fractures in adults (1) and may result from direct or indirect injuries. Usually, a direct injury typically causes a comminuted patellar fracture, while an indirect injury may lead to a transverse patellar fracture. Moreover, patellar fracture can present more fracture patterns due to the mechanism of combined injuries. The main objective behind the treatment of patellar fracture includes a restoration of the following: bone integrity, articular congruity of the patella, and also the function of the extensor mechanism.

The surgical indications for patellar fracture include a fracture separation greater than 2–4 mm, an articular incongruity greater than 2–3 mm, an incompetent extensor mechanism, and intra-articular loose bodies (2). Open reduction with internal fixation via the midline longitudinal incision over the patella is the most common treatment option for patellar fractures (3). Other surgical approaches include the median parapatellar approach and the lateral parapatellar approach (4, 5). Although these open surgical approaches can provide a good visualization of the patella and subsequently minimize the difficulty involved in the technique of reduction of bone fracture, they have some disadvantages such as surgical-related soft tissue injuries, destruction of the patellar blood supply, and reduced patient satisfaction due to the generation of a long surgical scar in front of the knee.

To overcome these drawbacks of open surgeries, minimally invasive surgeries with the advantages of fast recovery, few soft tissue injuries, and smaller surgical scars are being attempted and they are becoming increasingly popular. Some minimally invasive surgeries for patellar fractures have been reported recently. Shao et al. introduced a minimally invasive surgical technique for fixing a transverse patellar fracture by using the Zimmer® Cable-Ready® Cable Pin System (6). They found that minimally invasive surgeries produced better clinical outcomes in terms of knee function and pain in comparison with open surgeries. Akhilesh et al. performed a percutaneous tension-band wiring technique for patellar fractures. Although percutaneous tension-band wiring is a viable option for transverse patellar fractures, it is a technical challenge for the orthopedic surgeon (7).

In this study, we introduce a minimally invasive surgical approach, in which two 2 cm–3 cm paralleled incisions approximately 1 cm above the superior pole and 1 cm below the inferior pole of the patella are made to perform reduction and fixation, respectively, for patellar fractures. As the incision locates in the two poles of the patellar, we would like to call it a “bipolar incision” in brief. This study aims to introduce the bipolar incision method and assess the clinical outcomes of patellar fractures treated via this method.

## Materials and methods

### Patient selection

Patients who suffered patellar fractures and who received surgical treatment between 2018 and 2020 in our hospital were retrospectively reviewed. The ethics committee of our hospital approved the study protocol (approval number: [2015]084-1). The study was performed according to the principles outlined in the Declaration of Helsinki. Informed consent was obtained from all of the study participants. The indications for the bipolar incision were a transverse patella fracture with a displacement of >2 mm; the fracture could be fixed with a tension band and with Kirschner wires and a reduction of the displacement could be achieved by making an incision, with the surgeon’s experience also being a major factor in this. To reflect the true outcome of surgery, only those patients with normal knee function prior to injury and fresh closed fractures were included. Thus, the inclusion criteria were as follows: unilateral patellar fractures that were treated via bipolar incision; the knee function should be normal before the injury, with no other combined injuries; the technique of fixation should be tension-band wiring; there should be at least a 1-year follow-up. The exclusion criteria were as follows: open fracture; old fracture >2 weeks; a previous history of knee surgeries; complications arising from disease conditions, such as liver or renal failure, immunodeficiency or coagulation dysfunction and so on, which may affect the clinical outcomes of patellar fracture surgery.

### Surgical procedure and postoperative rehabilitation

**Figure 1:**
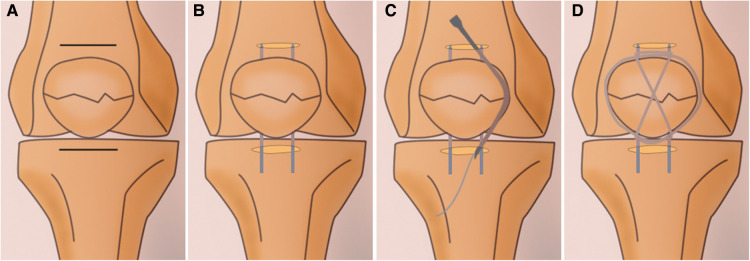
Diagrams show the main surgical procedures of the minimally invasive surgical technique. (**A**) Two paralleled incisions approximately one centimeter above and below the patella are made. (**B**) After a closed reduction of the fracture, two Kirschner wires are passed through the fracture fragments from the incision. (**C**) A wire passer or lumbar puncture needle is utilized to introduce the titanium cable subcutaneously. (**D**) The outcome of the fixation.

**Figure 2:**
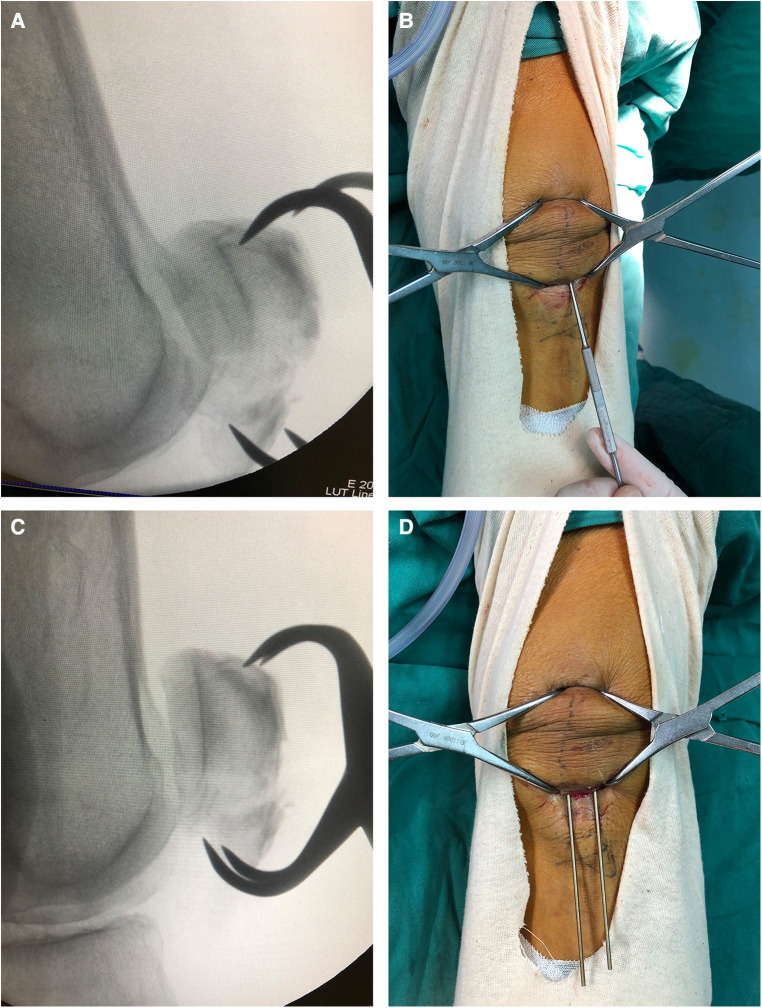
Joint application of periosteal elevator and pointed bone forceps helps achieve bone fracture reduction. (**A**) Radiograph taken after the initial reduction procedure reveals that fracture reduction was not achieved as the articular step-off still presented. (**B**) Photograph shows that a small periosteal elevator is passed through the patellar tendon to reach the articular surface of the distal bone fragments and elevate it, and then, two-pointed bone forceps were utilized to close the fracture gap. (**C**) Radiograph taken after the reduction procedure confirmed that the fracture was reduced. (**D**) Photograph shows two k-wires passing through the fracture to maintain the fracture reduction.

**Figure 3:**
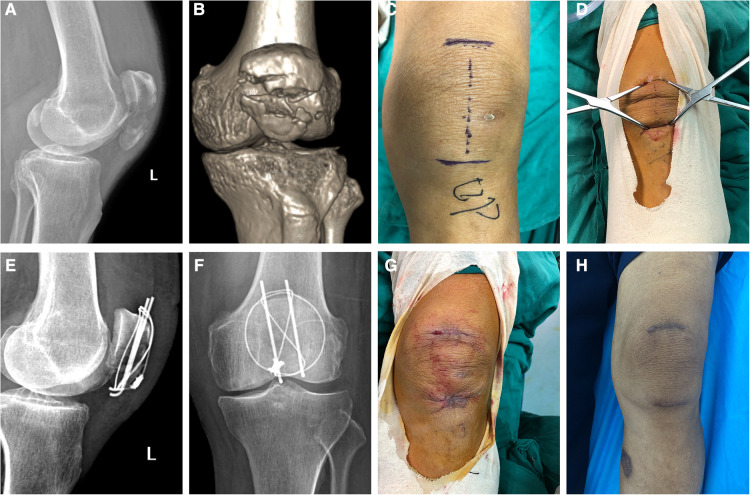
Radiographs and photographs of the knee of a 73-year-old male patient who suffered patellar fracture and received surgical treatment via the minimally invasive technique. (**A**) Lateral view of a preoperative radiograph of the knee joint. (**B**) 3D reconstruction image of patellar computer tomography scanning. (**C**) Photograph of an incision mark. (**D**) Two-pointed bone reduction forceps were applied to reduce the fracture displacement. (**E**) Lateral view of a postoperative radiograph of the knee joint reveals good bone reduction. (**F**) Anteroposterior view of a postoperative radiograph of the knee joint. (**G**) Photograph of an incision taken immediately after surgery. (**H**) Photograph of an incision taken in the third month after surgery.

**Figure 4:**
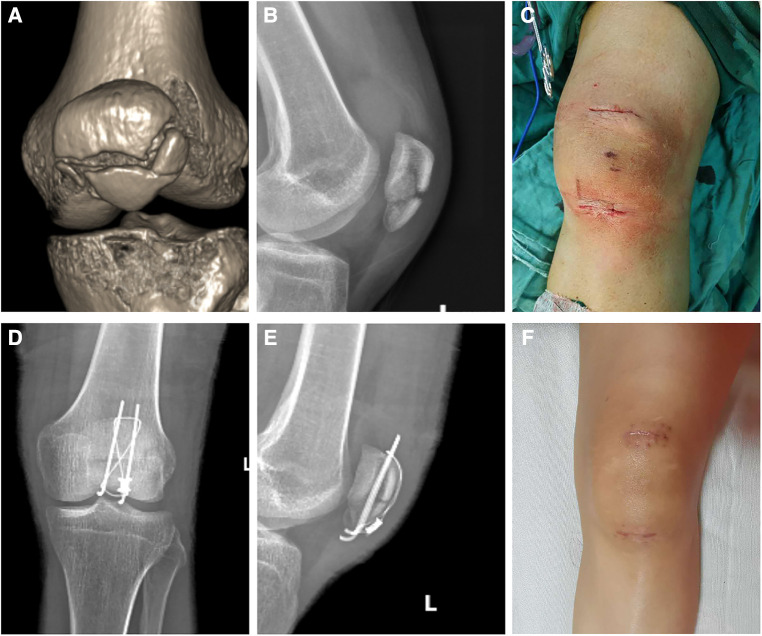
A 32-year-old female patient suffered patellar fracture in a traffic accident and was operated by using the minimally invasive technique. (**A**) 3D reconstruction image of patellar computer tomography scanning shows the patellar fracture. (**B**) Lateral view of a preoperative radiograph of the patella. (**C**) Photograph of an incision taken immediately after surgery. (**D**) Anteroposterior view of a postoperative radiograph of the knee joint. (**E**) Lateral view of the same radiograph shows anatomic reduction. (**F**) Photograph of an incision taken in the fourth month after surgery.

The surgical procedures were as follows. After spinal or general anesthesia, the patients were placed on the operating table. A pneumatic tourniquet was routinely applied at the base of the thigh. After sterilization of the leg, two 2 cm–3 cm paralleled incisions approximately 1 cm above the superior pole and 1 cm below the inferior pole of the patella were made to expose the quadriceps and patellar ligament (Figures 1A,B). Two two-pointed bone forceps were utilized to close the fracture gap from the medial and lateral sides of the patella. If the articular step-off was not well reduced, a small periosteal elevator was used to facilitate the reduction of bone fracture via insertion through the patellar ligament or the quadriceps to reach the articular surface of the bone fragments (Figure 2). After the closed reduction of the fracture, two Kirschner wires were passed through the fracture fragments from the incision. Two longitudinal incisions in the quadriceps were made around the two K-wires and extended to the superior pole of the patella. Also, the other two longitudinal incisions were made in the patella tendon around the K-wires and extended to the inferior pole of the patella, as described by Yan et al. (8). A wire passer or lumbar puncture needle was utilized to introduce the titanium cable subcutaneously. One titanium cable (Cable Grip System, Zimmer) was passed through the quadriceps and patellar tendons and behind the K-wires to form a circle and a figure 8, as shown in Figures 1C,D and Figure 3. In some non-comminuted transverse patellar fractures, the cable was only crossed over the front of the patella to form a figure 8, while the additional circle was not needed as shown in Figure 4. After the cable was locked, the Kirschner wire was cut and bent. The incisions in the quadriceps, patellar tendons, and skin were closed.

After closed reduction and stable fixation of the patellar fracture, the patients were encouraged to begin progressive exercises. On the first day after surgery, the patients were allowed to do quadriceps isometric exercises and passive flexion of the knee joint at 30°. On postoperative days 2–5, straight-leg-raising exercises were performed by the patients and also active flexion and extension of the knee joint was done to reach a 90° range of motion (ROM). Active knee exercises were continued until the recovery of knee function.

### Clinical and radiological assessment

The clinical and radiological records of all patients were reviewed. The mechanisms of injuries were analyzed. The classification of the patellar fracture was done based on the AO/OTA fracture and dislocation classification compendium-2018. The operation time, injured side, and blood loss during the operation were recorded. The Visual Analog Scale (VAS) scores of the knee at 45°and 90° flexion at postoperative day 5 and day 10 were assessed. The ROM and the modified Hospital for Special Surgery (HSS) knee score of the knee joint were assessed 1 year after surgery. At each outpatient visit, the patients were assessed clinically and radiographically. Radiographic union was defined as the disappearance of the fracture line, which was determined by the surgeons. Wound infection, skin flap necrosis, and failure of internal fixation were recorded.

### Statistical analysis

Statistical analysis was performed using SPSS (SPSS Inc., Chicago, IL, USA). Data were shown as means ± standard deviation. Owing to the retrospective and observational nature of this study, all data included in the study must be seen as only explorative and descriptive.

## Results

### Preoperative assessment

A total of 19 patients were included in this study. The demographic data of the patients are given in Table 1. The average age of the patients was 54.9 ± 16.4 years. There were 10 male and 9 female patients. A total of 8 fractures occurred on the left patella, while there were 11 fractures on the right patella. There were 12 cases of falls, while 7 were traffic accident cases. The average time for surgery after fracture was 3.4 ± 1.5 days. According to the AO/OTA fracture and dislocation classification compendium-2018, 11 patellar fractures were classified as 34C1 and 8 were classified as 34C2.

**Table 1:** Patient demographics and intraoperative data.

Parameter	*N* = 19
**Sex**	
Male	10
Female	9
Average age (years)	54.9 ± 16.4
**Cause of injury**	
Fall	12
Traffic accidents	7
**Classification**	
34C1	11
34C2	8
Mean time of operation (min)	69.0 ± 8.5
Average volume of blood loss (ml)	50.5 ± 11.8

### Intraoperative situation

All 19 patients were treated via the minimally invasive surgical approach. Separation of the bone fractures could usually be reduced through a closed reduction technique by using two-pointed forceps. While in some cases, articular step-off could not be reduce with the use of two-pointed forceps, as shown in Figure 2A, a small periosteal elevator proved helpful in the reduction, as shown in Figures 2B–D. The two transverse incisions could provide sufficient exposure for the fixation of tension-band wiring. The mean time of the operation was 69.0 ± 8.5 min, and the average volume of blood loss was 50.5 ± 11.8 ml.

### Radiographic and functional outcomes

All patients underwent a regular follow-up in the clinic. The mean time of the follow-up was 17.1 ± 4.0 months. Postoperative radiographs confirmed that each patellar fracture was well reduced and properly fixed. No implant failure such as breakage of the titanium cable or loosening of Kirschner wires, was observed at the radiographic follow-up. The radiograph of the last follow-up revealed that bone fractures were healed and the fracture lines disappeared in all patients. The mean time to union was 12.8 ± 2.1 weeks. The VAS score, HSS score, and the knee range of motion assessment are summarized in Table 2.

**Table 2:** Postoperative pain and knee function assessment.

Parameter	*N* = 19
^a^ **VAS score/5 days**	
45°	4.3 ± 0.9
90°	5.9 ± 0.8
**VAS score/10 days**	
45°	3.1 ± 0.8
90°	4.4 ± 0.7
HSS	97.1 ± 2.6
**Total Knee** ^a^**ROM (°)**	
2 weeks ^a^PO	96.1 ± 7.8
12 weeks PO	120.5 ± 5.6
1 years PO	131.8 ± 4.4

^a^ ROM, range of motion; VAS, visual analog scale; PO, postoperation.

### Complications

No surgical-related complications such as neurovascular injuries, wound infection, or cutaneous necrosis occurred perioperatively. All fractures healed successfully, and no implant failure was observed.

## Discussion

The patella, as the largest sesamoid bone in the body, plays an important role in increasing the lever arm of the quadriceps extensor mechanism. A patellar fracture can be caused by both direct trauma and an indirect traumatic mechanism. Numerous fixation techniques and materials have been applied for patellar fractures that require surgery, such as tension bands, cerclage wiring, suture repair, and osteosynthesis with plates and screws. The most frequent patellar fractures are transverse fractures (6, 9). Open reduction and internal fixation with tension bands and Kirschner wires is still the most widely used technique for repairing transverse fractures (10–12). Nevertheless, the classic open approach may lead to a certain amount of wound complications, which include postoperative adhesions, wound infections, and non-cosmetic scars (13, 14). To overcome these drawbacks of the open approach, we treated the patellar fractures in our patients through a more minimally invasive approach, which was by making two paralleled incisions above and below the patella. We could perform the techniques of reduction of fractures as well as internal fixation by placing a tension band and Kirschner wires on the two skin windows successfully. The incision was in the same direction as the skin texture and was smaller than the classic open approach. Therefore, the incision led to the formation of only small scars in front of the knee, which resulted in improved patient satisfaction. Postoperative pain and knee function assessment revealed that the outcome of the patellar fractures treated via the new approach was favorable.

Minimally invasive surgical techniques are becoming increasingly popular for the treatment of traumatic injuries (15, 16). These methods have the advantages of causing decreased injuries to the soft tissue, preserving blood supply, producing only cosmetic surgical scars, and ensuring speedy recovery. Many minimally invasive techniques have been reported for the treatment of patellar fractures. Rathi et al. (7) introduced a percutaneous tension-band wiring technique for such fractures. Both reduction and fixation of fractures were performed percutaneously. Although it was a viable option, it was technically challenging, because the procedure of cutting and bending Kirschner wires as well as burying the knot might cause difficulties for the surgeon. Vicenti et al. (13) managed transverse patellar fractures in elderly patients with a minimally invasive osteosynthesis technique. In their surgical procedure, a 1 cm incision was made at the superolateral border of the patella to introduce steel wires, and other procedures were performed percutaneously. The additional incision could reduce the difficulty of surgery. Shao et al. (6) also reported their work on the treatment of patellar fractures via a minimally invasive surgical technique and fixation with the Cable Pin System and found that this surgical technique could provide better clinical results and improved knee function than open surgery. Although many studies concluded that minimally invasive techniques had better clinical outcomes, the limited exposure in such methods usually led to increased technical difficulties during surgery and restricted their wide application. Therefore, it became necessary to find a way to repair fractures with a minimally invasive incision but also with one that caused limited problems. Although the bipolar incision technique resulted in larger scars than the complete percutaneous technique and the semipercutaneous technique introduced by Shao et al. (6), it could expose the inferior and superior poles of the patella and facilitate the reduction procedure with two-pointed forceps. In addition, it could make the process of cutting and bending Kirschner wires easier so as to avoid leaving too long wires in the soft tissue, which may potentially lead to postoperative soft tissue irritation. In this study, none of the patients encountered hardware related complications such as irritation of the skin, as raised by Vicenti et al. (13), who carried out surgery through the percutaneous technique. Two incisions reduced the technical difficulty of the surgery, this incision was applicable for the treatment of a more complicated patellar fracture displacement.

Although the tension-band wiring technique is the most common fixation technique for patellar fracture, the impact of technical issues on the outcome cannot be ignored. The interposition of soft tissue between the patella and the tension band may weaken the compression force and lead to a higher failure rate (17). Additional incision-making in the patellar tendon and the quadriceps tendon of the upper and lower poles of the patella where the Kirschner wire passes through can help eliminate the soft tissue interposition between the titanium cable and the bone, which will subsequently improve the clinical outcomes and knee function (8). In the current surgical approach, the two incisions could provide sufficient exposure to perform the above procedures to place the tension-band wire as close as possible to the bone. Moreover, although a figure-of-8 fashion tension-band wire was adequate for most transverse patellar fractures, the modified tension-band technique, which is a combination of cerclage and the tension band, could provide a greater strength of fixation and allow early mobilization (8, 18, 19). By using a lumbar puncture needle, we could introduce the titanium cable subcutaneously to do the modified tension-band fixation for a comminuted patellar fracture. Thus, this minimally invasive surgical approach was feasible to make an ideal tension-band fixation for patellar fracture.

Bone fractures could not be visualized directly through the minimally invasive surgical approach. Thus, sufficient preoperative assessment of the bone fracture was essential. Anteroposterior x-ray radiograph was of only limited value for making a judgment on bone fracture displacement as the patella is overlapped with the femur. Three-dimensional reconstruction images of computerized tomography could help us understand the patellar fracture better and was useful for preoperative planning. An intraoperative x-ray radiograph was routinely applied to view the reduction and fixation of fractures. Nevertheless, arthroscopy could help make a more precise evaluation of fragment reduction (20). Although in some patients, the primary reduction of the bone fracture using two-pointed forceps failed, the reduction could finally be achieved by using the Kirschner wire joy-stick technique or a mall periosteal elevator.

This study had some limitations. The fracture displacements of all cases were finally reduced successfully. No failed fracture reduction was observed which might be due to the small sample size. Thus, experience in the management of patients with failed fracture reduction could not be provided. Patients with failed fracture reduction might need the assistance of arthroscopy based on literature potentially. No complications could be identified due to the small sample size and short follow-up time. Thus, going forward, longer follow-ups are required. No control group was included in this study. Therefore, we should not attribute the good functional outcome solely to the new surgical approach. Nevertheless, our findings showed that the minimally invasive surgical approach presented in this study was a feasible method for treating patella fractures.

To sum up, the minimally invasive bipolar incision method could decrease the technical difficulties associated with minimally invasive surgery for patellar fractures. This type of incision could provide sufficient exposure for fracture reduction and internal fixation. The favorable clinical outcomes revealed that the bipolar surgical approach was a feasible method for repairing patellar fractures.

## Data Availability

The raw data supporting the conclusions of this article will be made available by the authors without undue reservation.
